# Improved survival in pediatric acute lymphoblastic leukemia through therapy intensification based on minimal residual disease and protocol-driven early response risk classification

**DOI:** 10.1007/s44313-025-00085-3

**Published:** 2025-07-09

**Authors:** Hyery Kim, Su Hyun Yoon, Sunghan Kang, Kyung-Nam Koh, Ho Joon Im, Daehyun Chu, Mi Young Kim, Young-Uk Cho, Sang-Hyun Hwang, Seongsoo Jang

**Affiliations:** 1 Department of Pediatrics, University of Ulsan College of Medicine, Asan Medical Center, Seoul, South Korea; 2 Department of Laboratory Medicine, University of Ulsan College of Medicine, Asan Medical Center, Seoul, South Korea

**Keywords:** Acute lymphoblastic leukemia, Children, Minimal residual disease

## Abstract

**Purpose:**

Minimal residual disease (MRD)-guided therapy is the global standard treatment for pediatric acute lymphoblastic leukemia (ALL). We assessed the impact of MRD-driven intensification along with protocol-defined risk groups in pediatric ALL treatment.

**Methods:**

This retrospective analysis included 209 patients with ALL (treated between January 2013 to June 2023). MRD was assessed using six- to eight-color flow cytometry at the end of each phase before the maintenance phase. Post-induction treatment was determined based on early response, National Cancer Institute risk, and cytogenetics. High-risk (HR) patients followed the Korean HR or CCG-1882 protocols and standard-risk (SR) patients followed the modified COG-AALL0331 protocol. Treatment was intensified if flow-MRD ≥ 0.1% was identified.

**Results:**

Overall, 103 and 106 patients were classified as having SR and HR, respectively. The 5-year overall survival (OS) and event-free survival (EFS) were 92.5% and 84.3%, respectively. Thirty SR and 18 HR patients received intensified chemotherapy. Treatment intensification significantly improved EFS in patients with high MRD (94.2% vs. 75.5%, *p* = 0.04), particularly in post-induction patients with high MRD (90.0% vs. 19.0%, *p* = 0.035). The difference in survival between rapid early responder (RER) and slow early responder (SER) groups was eliminated after MRD-based intensification. The implementation rates of treatment intensification varied over time (9.1% before 2015, 28.6% during 2016–2019, and 13.9% during 2020–2023), reflecting improved risk stratification and therapy selection.

**Conclusion:**

MRD-guided therapy intensification markedly improved survival outcomes in patients with pediatric ALL when combined with risk-based protocols, highlighting the importance of MRD monitoring for optimizing risk-adapted treatment strategies.

**Supplementary Information:**

The online version contains supplementary material available at 10.1007/s44313-025-00085-3.

## Introduction

Acute lymphoblastic leukemia (ALL) is the most common childhood cancer, accounting for approximately 25% of all pediatric malignancies [1]. Despite notable advancements in ALL therapy, which have markedly enhanced long-term survival rates to more than 90%, treatment outcomes vary extensively based on biological and therapeutic factors [2]. A pivotal development in this regard is the assessment of minimal residual disease (MRD) to guide treatment decisions [3].

MRD refers to a small number of leukemic cells that persist during and beyond treatment, despite the absence of disease detection using conventional diagnostic methods [4]. The prognostic relevance of MRD in pediatric ALL has been well-documented, with various studies demonstrating its predictive value for relapse and overall survival (OS) [3–5]. Consequently, MRD measurements have become an integral part of risk stratification and treatment modulation in contemporary ALL treatment protocols.

The traditional risk classification for ALL treatment initially involves a combination of factors, including age, white blood cell (WBC) count at diagnosis, cytogenetics, and response to initial chemotherapy, termed early morphologic response [2]. This initial stratification allows oncologists to categorize patients into standard-risk (SR) and high-risk (HR) cohorts, dictating subsequent therapy. However, the integration of MRD assessment into this framework offers a more precise method for reassessing and potentially recalibrating treatment intensity during therapy [5, 6].

Advances in MRD technology have expanded from conventional flow cytometry, known as flow-MRD, to include more sensitive and specific methods, such as next-generation sequencing (NGS) [4]. However, flow-MRD faces barriers to inclusion in prospective multicenter protocols owing to challenges in standardization across centers and its low sensitivity in distinguishing regenerating cells or hematogones from leukemic blasts [4]. Recently, NGS-MRD testing has facilitated higher resolution of residual disease detection, enabling the identification of leukemic clones that may evade flow cytometric detection [7]. The transition to NGS-MRD in clinical settings, as evidenced in August 2021 in South Korea, reflects the ongoing evolution towards more accurate and informative diagnostic modalities that can substantially influence treatment decisions.

In this study, we aimed to assess the efficacy of flow-MRD-based therapy by comparing the treatment outcomes between patients who received therapy based on a traditional protocol-based risk classification without flow-MRD and those who received intensified therapy according to flow-MRD.

## Materials and methods

### Data source and study population

The target participants were patients diagnosed with precursor ALL, who were treated at the Seoul Asan Medical Center Children's Hospital between January 2013 and June 2023. Initial chemotherapy followed three risk group classifications at diagnosis: SR, HR, and very HR (VHR). The SR group comprised patients with 1 ≤ age < 10 years at diagnosis, WBC count less than 50 × 10^9^/L, and no molecular cytogenetic risk factors. Patients were assigned to the HR group if they had a WBC count ≥ 50 × 10^9^/L, age ≥ 10 years, or T-cell immunophenotype. Patients with Philadelphia chromosome or *BCR::ABL* rearrangement, chromosomes less than 45 as determined by cytogenetics, or t(4:11) abnormalities in leukemia blasts were assigned to the VHR group. This study excluded infants with ALL or patients classified as VHR who underwent hematopoietic stem cell transplantation at the time of the first complete remission (CR). The medical records of the patients were retrospectively reviewed.

In the SR group, the treatments followed the modified COG-AALL0331 protocol (Supplementary Table 1). Based on the results of bone marrow (BM) examinations conducted on days 8 and 15 following induction, if the patient attained CR, they were classified as slow early responders (SER: M2 or M3 at day 15 BM) or rapid early responders (RER: M1 at day 8 or day 15 BM). The planned treatment for the RER group included intensified consolidation, standard interim maintenance (IM), standard delayed intensification (DI), and maintenance. Planned treatments for the SER group included enhanced consolidation, augmented IM #1, augmented DI #1, augmented IM #2, augmented DI #2, and maintenance.

 Before March 2015, the HR group initially underwent treatment according to the Korean multicenter study 0601 protocol, followed by treatment according to the Korean multicenter study 1501 protocol (Supplementary Table 1). The HR criteria for 1501 regimen were the same as those used for 0601 regimen except that a WBC count ≥200 × 10^9^/L was an exclusion criterion in 0601 but not in 1501, which also included patients with CNS or testicular leukemia. In the 0601 protocol, treatment after consolidation varied based on early response. For patients with rapid early response (RER; defined as M1 or M2 marrow on day 8), a single interim maintenance (IM) and single delayed intensification (DI) were administered. In contrast, for patients with slow early response (SER; defined as M3 marrow on day 7 or extramedullary involvement), double IM and double DI were given, followed by maintenance therapy. During the second DI, patients with CNS3 status received craniospinal radiotherapy (cranial irradiation 1800 cGy plus spinal irradiation 600 cGy), while other SER patients received prophylactic cranial irradiation at 1200 cGy. In 1501 protocol, the RER (M1 or M2 at day 8 and M1 at day 15) group was treated with 1st IM-DI-2nd IM-maintenance after consolidation; the SER (other than RER, extramedullary involvement, WBC ≥ 100,000/µL) group was treated with 1st IM-1st DI-2nd IM-2nd DI-maintenance. In the 1501 protocol, RER was defined more strictly (M1 or M2 on day 8 and M1 on day 15), and those patients received 1st IM–DI–2nd IM followed by maintenance therapy after consolidation. The SER group (defined as not meeting RER criteria, or having extramedullary involvement or WBC ≥100 × 10^9^/L) received 1st IM–1st DI–2nd IM–2nd DI followed by maintenance. The 1501 regimen did not involve preventive cranial irradiation; craniospinal radiotherapy (cranial irradiation 1800 cGy + spinal irradiation 600 cGy) was performed only in patients with CNS3 status.

### MRD assessment

A MRD analysis was conducted after each treatment cycle. For MRD detection, flow cytometric analysis was performed using a combination of monoclonal antibodies based on cross-lineage antigen expression observed at diagnosis. MRD analysis was performed using six- to eight-color-based flow cytometry as described previously [4, 8]. From August 2021, the analysis was performed using NGS; however, for comparison purposes, both flow-MRD and NGS-MRD were conducted concurrently with treatment intensification decisions based solely on the flow-MRD.

### Treatment modification according to MRD

Cases with a flow MRD level of 0.1% or higher were classified as "High-MRD" and analyzed. If flow-MRD of ≥ 0.1% was detected after each phase, treatment was intensified in certain patients from SR-RER to SR-SER, SR-SER to HR-SER, or HR-RER to HR-SER. If a patient was originally allocated to HR-SER and exhibited high MRD, extra ifosfamide (1.8 gm/m^2^ in DNK2 225 mL/m^2^ IV over 90 min, D1-5 with Mesna) and etoposide (100 mg/m^2^ in NS 300 mL/m^2^ IV over 2 h, D1-5), along with intrathecal methotrexate, were added immediately after the treatment phase in which high MRD was detected.

### Statistical analysis

Descriptive statistics were used to analyze patient characteristics. An event was defined as a relapse, secondary malignancy, or death from any cause. Survival probabilities were estimated using Kaplan–Meier plots, and the log-rank test was used to assess the equality of survival functions across groups. Statistical significance was set at *p* < 0.05. Statistical analyses were performed using IBM SPSS Statistics (version 29.0; IBM Corp., Armonk, NY).

## Results

### Characteristics of the patients

Of the 209 patients included in the analysis (Table 1), 103 were classified as SR and 106 as HR. The median age at diagnosis was 4.6 and 10.5 years, respectively. The T-cell immunophenotype was detected in 14 patients. According to the molecular genetic classification, B-ALL and not otherwise specified (NOS) were the predominant types in both groups, with B-ALL featuring *ETV6::RUNX1* observed in the SR group and B-ALL with *E2A::PBX1* in the HR group. All patients with SR received treatment based on the modified COG-AALL0331 regimen. Conversely, 21 HR patients were treated according to the 0601 protocol, while 85 HR patients received treatment according to the 1501 protocol. At diagnosis, the percentage of patients with CNS involvement was 4.9% and 6.6% in the SR and HR groups, respectively.

**Table 1:** Characteristics of patients by NCI risk groups

		Standard Risk		High Risk	
		*N* = 103		*N* = 106	
Age	median (range, years)	4.6 (1.1–9.7)		10.5 (1.2–17.3)	
WBC	median (range, /µL)	6600 (600–47900)		40700 (500–492100)	
Sex	Female	53	51.5%	43	40.6%
	Male	50	48.5%	63	59.4%
Immunophenotype	B-cell	103	100%	92	86.8%
	T-cell	0	0.0%	14	13.2%
Molecular genetics	B lymphoblastic leukemia/lymphoma, NOS	30	29.1%	39	36.8%
	B lymphoblastic leukemia/lymphoma with t(1;19)(q23;p13.3); *E2A::PBX1 *(*TCF3::PBX1*)	4	3.9%	20	18.9%
	B lymphoblastic leukemia/lymphoma with t(12;21)(p13;q22); *TEL::AML1* (*ETV6::RUNX1*)	23	22.3%	8	7.5%
	B lymphoblastic leukemia/lymphoma with *FUS::ERG*	0	0.0%	2	1.9%
	B lymphoblastic leukemia/lymphoma with hyperdiploidy	43	41.7%	19	17.9%
	B lymphoblastic leukemia/lymphoma with iAMP21	0	0.0%	1	0.9%
	B lymphoblastic leukemia/lymphoma with t(v;11q23); MLL rearranged	3	2.9%	3	2.8%
	T lymphoblastic leukemia/lymphoma, *STIL::TAL1*	0	0.0%	7	6.6%
	T lymphoblastic leukemia/lymphoma, NOS	0	0.0%	7	6.6%
Chemotherapy regimen	modified COG-AALL0331	103	100%		
	Korean multicenter study-0601			21	19.8%
	Korean multicenter study-1501			85	80.2%
CNS status at diagnosis	1	96	93.2%	96	90.6%
	2	2	1.9%	3	2.8%
	3	5	4.9%	7	6.6%
Induction failure		2	1.9%	3	2.9%
Protocol-based treatment stratification at the end of induction		RER	SER	RER	SER
		80	21	50	53
*Modified treatment based on the treatment response*	*0331 RER*	*54*	*1*		
	*0331 SER*	26*	*16*		
	*0601 RER*			*7*	*3*
	*0601 SER*			3*	*6*
	*1501 RER*			*25*	*3*
	*1501 SER*		4*	15*	*41*
Protocol-based treatment (no intensification)		71	68.9%	90	84.9%
	EOI MRD high	2		9	
	EOC MRD high	21		21	
	Post-phase MRD high	6		7	
Treatment intensification done		30	29.1%	18	12.3%
*Cause of intensification*	Very slow induction response	6		2	
	EOI MRD high	7		5	
	EOC MRD high	9		7	
	Post-phase MRD high	6		3	
	No further use of asparaginase d/t pancreatitis	2			
	Complex chromosome			1	

* Abbreviations* *EOI* end of induction, *EOC* end of consolidation, *MRD* minimal residual disease, *NCI* National Cancer Institute, *RER* rapid early responder, *SER* slow early responder

*treatment intensified patients

### Treatment stratification and modification based on MRD status

After induction, 101 patients (98.1%) in the SR group and 103 patients (97.1%) in the HR group achieved remission (Table 1). Upon classifying the patients into risk groups according to the protocol, 80 patients (79.2%) in the SR group were identified as having RER, whereas 21 patients (20.8%) were categorized as having SER. In the HR group, 50 patients (48.5%) were classified as RER, whereas 53 (51.5%) were classified as SER. Treatment was intensified in 30 (29.1%) and 18 (12.3%) patients in the SR and HR groups, respectively. The cause of intensification was high MRD at the end of induction (EOI MRD) in seven SR and five HR patients. In addition, the cause of intensification was high MRD at the end of consolidation (EOC MRD) in nine SR and seven HR patients. Moreover, the cause of intensification was high MRD assessed immediately following subsequent treatment phases after consolidation (post-phase MRD) in six SR and three HR patients. Irrespective of MRD, if both day 8 and day 15 BM results were M2 or higher, patients were considered to have a very slow induction response and underwent treatment intensification. There were six such patients in the SR group and two in the HR group.

Supplementary Fig. 1 presents a comprehensive flowchart summarizing patient classification based on MRD assessments at different treatment phases and subsequent treatment intensification decisions applied to the analytic cohort. In our analysis of the MRD pattern throughout the treatment period involving 204 patients who achieved remission, we observed that 49.5% (101 204) of the patients exhibited high MRD at least once during this period (Supplementary Table 2). Among the patients, 10.3% exhibited high EOI MRD, 32.8% had high EOC MRD, and 45.1% had high post-phase MRD. Only 2% of the patients exhibited high post-phase MRD (MRD assessed immediately following subsequent treatment phases after consolidation), indicating that the majority of patients who experienced high MRD at any point during treatment maintained high MRD through the completion of consolidation. In a cohort of 37 patients who received intensified treatment for elevated MRD, the analysis segmented into 4-year intervals revealed that 9.1%, 28.6%, and 13.9% of patients were classified in the treatment change group for the periods up to 2015, from 2016 to 2019 and from 2020 to 2023, respectively. This represented 17.2%, 51.2%, and 34.5% of all patients with high MRD, respectively, during each period.

### Survival results according to treatment intensification based on MRD

**Fig. 1:**
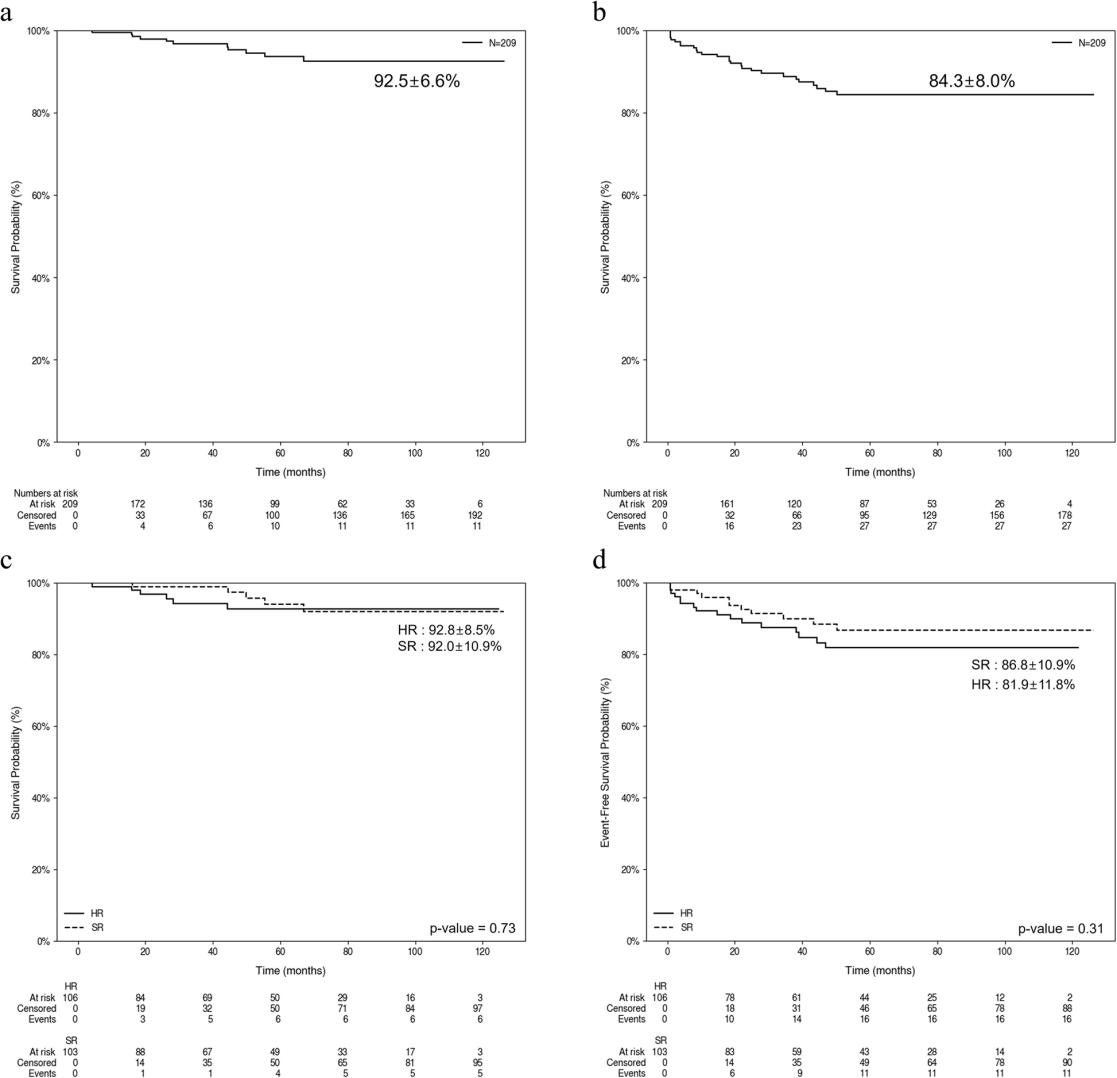
Survival outcome in all patients. **a** Kaplan–Meier curve showing overall survival (OS) for all patients. The estimated 5-year OS rate is 92.5 ± 6.6%. **b** Kaplan–Meier curve showing event-free survival (EFS) for all patients. The estimated 5-year EFS rate is 84.3 ± 8.0%. **c** Kaplan–Meier curves comparing overall survival between National Cancer Institute (NCI) high-risk (HR) and standard-risk (SR) groups. The 5-year OS rates are 92.8 ± 8.5% for HR patients and 92.0 ± 10.9% for SR patients (*p* = 0.73). **d** Kaplan–Meier curves comparing EFS between NCI HR and SR groups. The 5-year EFS rates are 81.9 ± 11.8% for HR patients and 86.8 ± 10.9% for SR patients (*p* = 0.31). **a**. Overall survival. **b**. Event-free survival. **c**. Overall survival by NCI risk groups. **d**. Event-free survival by NCI risk groups

The median follow-up duration for all patients was 59 months (range, 2–126 months). The five-year OS was 92.5 ± 6.6%, while the event-free survival (EFS) at the same interval was 84.3 ± 8.0% (Fig. 1a and b). At diagnosis, OS was 92.0 ± 10.9% and 92.8 ± 8.5% in the SR and HR groups, respectively, showing no statistically significant difference (*p* = 0.73) (Fig. 1c). EFS was also not significantly different between the SR and HR groups (*p* = 0.31) (Fig. 1d).

Comparison of EFS based on flow MRD levels before and after the induction phase revealed a statistically significant difference in survival rates associated with MRD (Supplementary Fig. 2).

**Fig. 2:**
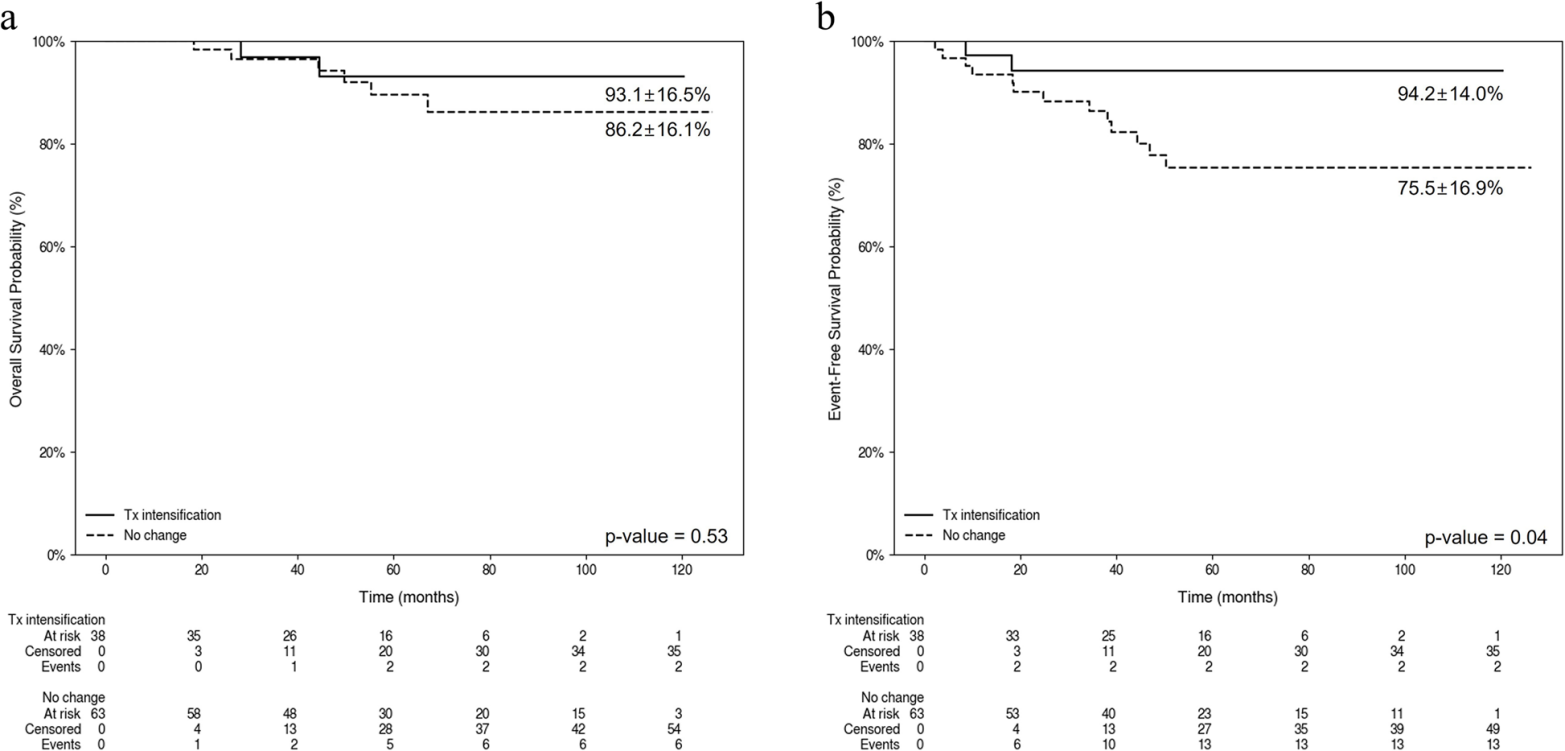
Survival rates according to treatment intensification in patients with high minimal residual disease (MRD). **a** Kaplan–Meier curve showing overall survival (OS) in patients with high MRD levels, stratified by treatment intensification. The estimated 5-year OS rate is 93.1 ± 16.5% for patients who received treatment intensification and 86.2 ± 16.1% for those without treatment changes (*p* = 0.53). **b** Kaplan–Meier curve showing event-free survival (EFS) in patients with high MRD levels, stratified by treatment intensification. The estimated 5-year EFS rate is 94.2 ± 14.0% for patients who received treatment intensification and 75.5 ± 16.9% for those without treatment changes (*p *= 0.04). **a**. Overall survival. **b**. Event-free survival

In a cohort of 101 patients exhibiting high MRD, no statistically significant differences in key characteristics were observed between the 38 patients who underwent treatment intensification and those 63 who did not (Supplementary Table 3). However, the 38 patients who underwent treatment intensification demonstrated a statistically significant improvement in EFS (Fig. 2b), but not in OS (Fig. 2a).

**Fig. 3:**
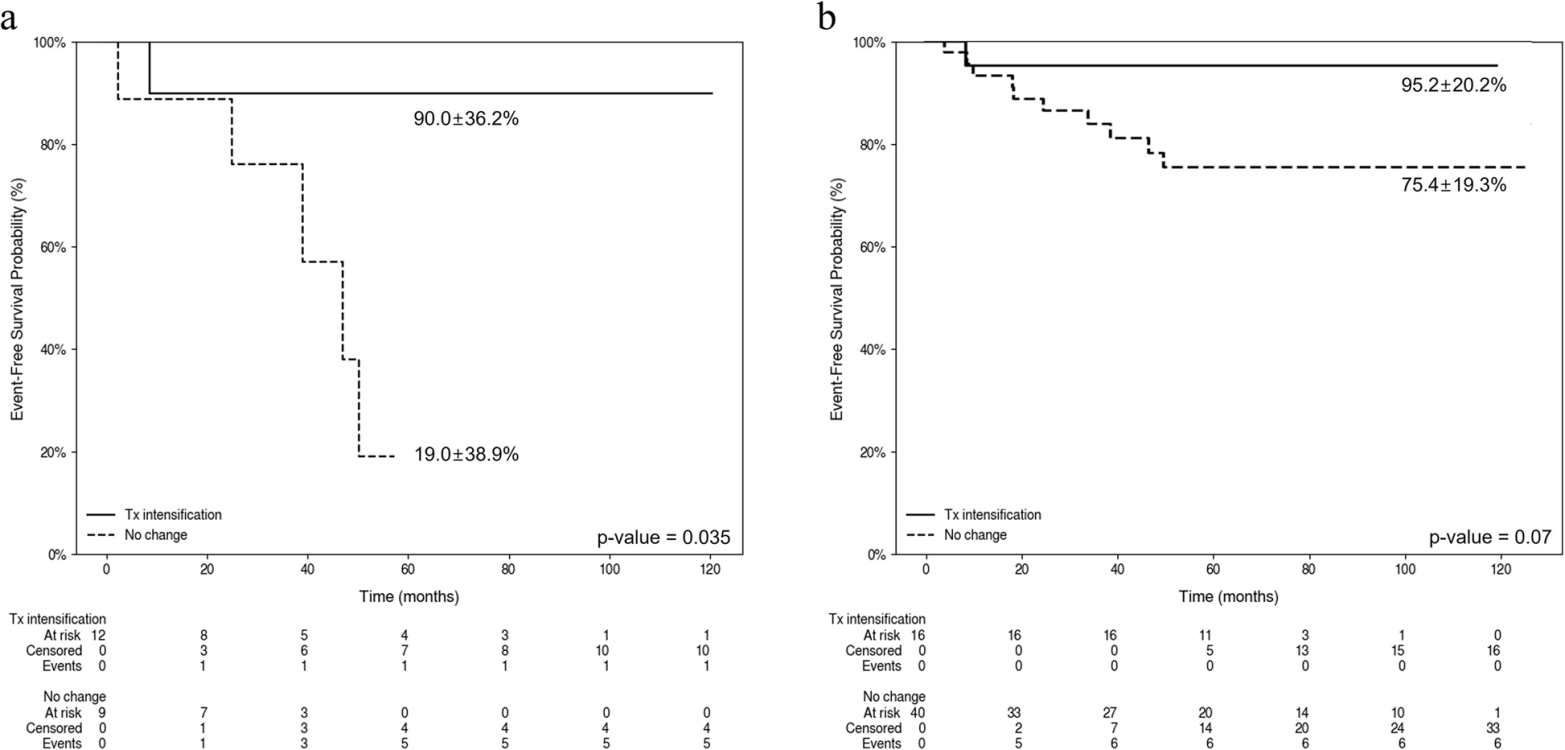
Survival rate according to treatment intensification based on the phase of high minimal residual disease (MRD). **a** Kaplan–Meier curve showing overall survival in patients with high MRD levels at the end of induction, stratified by treatment intensification. The estimated 5-year survival rate is 90.0 ± 36.2% for patients who received treatment intensification compared with 19.0 ± 38.9% for those without treatment changes (*p* = 0.035). **b** Kaplan–Meier curve showing overall survival in patients with high MRD levels at the end of consolidation, stratified by treatment intensification. The estimated 5-year survival rate is 95.2 ± 20.2% for patients who received treatment intensification and 75.4 ± 19.3% for those without treatment changes (*p* = 0.07). Patients with high MRD at the end of induction who underwent treatment intensification were excluded from this analysis; only patients with high MRD at the end of consolidation among the remaining cohort were included. **a**. Patients with high MRD at the end of induction. **b**. Patients with high MRD at the end of consolidation

We analyzed survival rates based on treatment intensification in relation to the high MRD observed after induction versus consolidation. Among patients exhibiting high EOI MRD, those who received treatment intensification demonstrated an EFS of 90%, which significantly surpassed the EFS of 19.0% observed in patients who did not receive intensification (Fig. 3a). In patients with high EOC MRD, EFS was generally higher among those who underwent treatment intensification; however, this difference was not statistically significant (Fig. 3b).

**Fig. 4:**
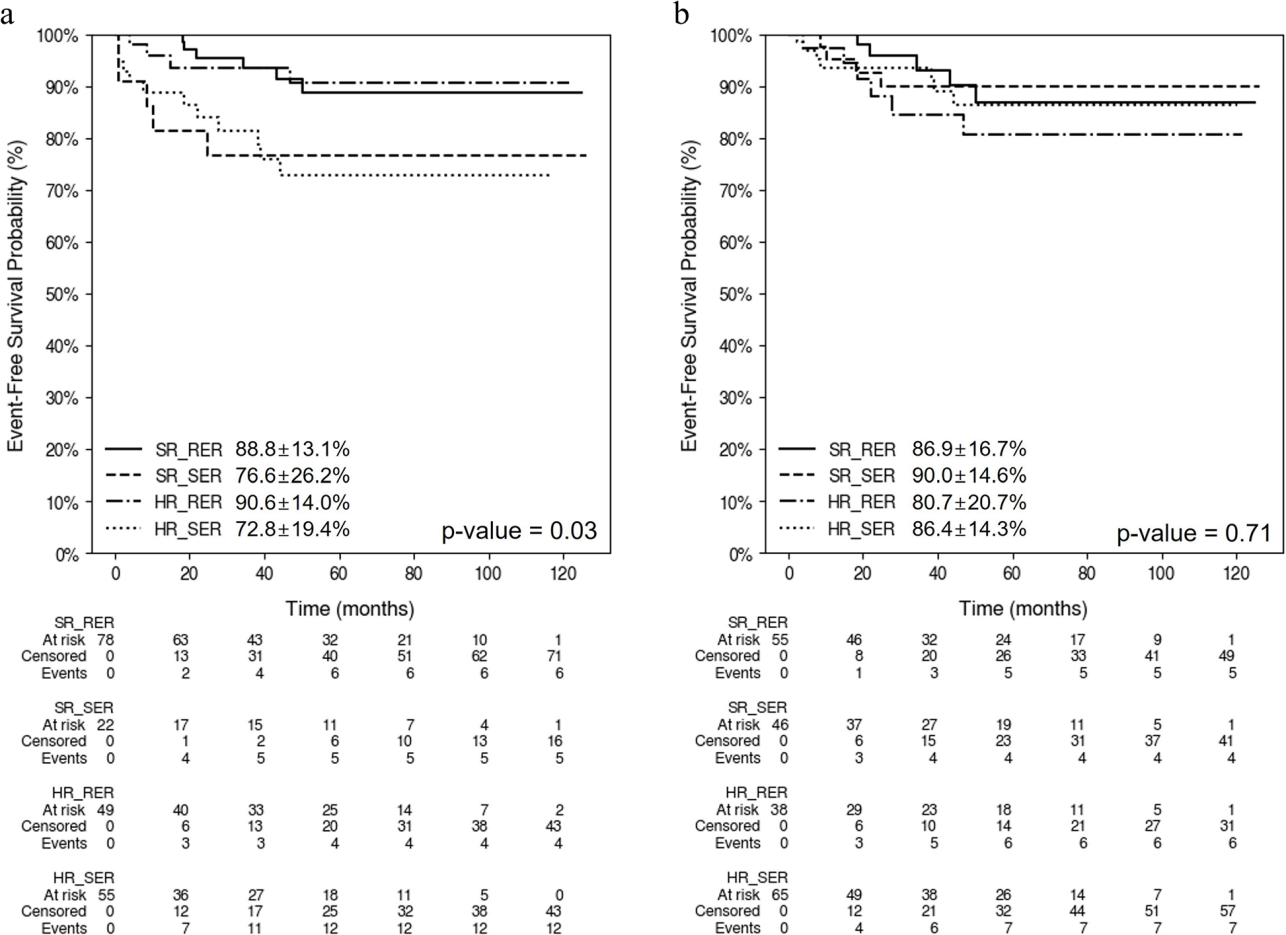
Event-free survival (EFS) rates in protocol-defined risk groups and those with treatment intensification based on minimal residual disease (MRD). **a** Kaplan–Meier curves depicting EFS according to the original protocol-defined risk groups. The 5-year EFS rates are 88.8 ± 13.1% for SR_RER, 76.8 ± 22.6% for SR_SER, 90.6 ± 14.0% for HR_RER, and 72.8 ± 19.4% for HR_SER (*p *= 0.03). **b** Kaplan–Meier curves depicting EFS according to modified risk groups after treatment intensification based on MRD levels. The 5-year EFS rates are 86.9 ± 16.7% for SR_RER, 90.0 ± 14.6% for SR_SER, 80.7 ± 20.7% for HR_RER, and 86.4 ± 14.3% for HR_SER (*p* = 0.71). **a**. EFS according to the protocol-defined risk group (original risk group). **b**. EFS according to the modified risk group, intensified based on the MRD. RER, rapid early responder; SER, slow early responder; HR, high risk; SR, standard-risk

Upon application of the original protocol that categorized patients into risk groups based on the induction response, both SR and HR patients exhibited reduced EFS in the SER patient group compared with that in the RER patient group (Fig. 4a). Upon analyzing EFS in relation to the risk groups following treatment intensification based on flow-based MRD, in conjunction with risk groups indicative of a morphological response during induction, we detected no statistically significant differences in survival rates across the risk groups (Fig. 4b).

## Discussion

Our findings revealed the substantial impact of MRD-guided therapy intensification combined with protocol-based risk stratification on survival outcomes in pediatric patients with ALL. Our analysis of 209 patients over a 10-year period revealed that MRD-guided treatment intensification markedly improved survival outcomes, particularly in patients with high EOI MRD.

The integration of MRD assessment has fundamentally altered risk stratification and therapeutic decision-making in ALL treatment [3]. In our study, flow cytometry analysis showed that 49.5% of the patients exhibited a high MRD at least once during treatment, with distinct temporal patterns: 10.3% at EOI, 32.8% at EOC, and 45.1% at the post phase after consolidation. Only 2% of patients exhibited high MRD solely after consolidation, suggesting that early MRD patterns highly predict the treatment response [6, 9].

The observed cumulative high MRD rate (MRD ≥ 0.1%) of 49.5% in our cohort reflects comprehensive multi-phase monitoring rather than discordance with prior literature. While earlier studies, such as COG AALL0232 (16.3% EOI) [6] and AIEOP-BFM 2000 (13.7% EOI and 13.8% EOC) [10], reported similar or lower rates at single time points as in our study (10.3% EOI and 32.8% EOC), these differences stem from methodological differences. Unlike studies focusing solely on induction- or consolidation-phase MRD, we evaluated MRD at the EOI (10.3%), EOC (32.8%), and post phases (45.1%), capturing late-emerging positivities. Unlike studies with fixed MRD assessment points, our MRD-guided algorithm prompted repeated testing whenever ≥ 0.1% was detected, increasing cumulative positivity capture. Furthermore, depending on whether a six-color or an eight-color panel is used, the sensitivity of the assay might vary. This variability in sensitivity, one of the major limitations of flow MRD, could have resulted in a higher MRD rate than the actual rate. It is anticipated that the adoption of NGS-MRD will resolve the issues associated with the unstable sensitivity of flow cytometric analysis.

When the original COG-AALL0331 protocol and Korean multicenter protocols were first initiated, MRD assessment methods were not yet standardized, and treatment stratification primarily relied on morphology-based early response evaluation [11]. During this period, our institution, like several others, utilized early morphological response criteria such as RER and SER for risk stratification. The transition from morphology-based risk stratification to MRD-guided therapy reflects the pivotal evolution in pediatric ALL treatment [12, 13].

Our data provide compelling evidence regarding the efficacy of MRD-guided treatment intensification. Patients who underwent intensified treatment based on high MRD had significantly better outcomes (94.2% vs. 75.5%, *p* = 0.04), with the most striking difference observed in patients with high MRD post-induction (90% vs. 19%, *p* = 0.035). These results align with findings from major cooperative group studies, including the AIEOP-BFM ALL 2000 study and COG and DCOG-ALL studies [10, 13, 14].

Analysis of survival rates between the protocol-defined risk groups and MRD-guided treatment intensification revealed particularly interesting patterns. Although the original risk stratification showed significant differences in EFS between the RER and SER groups, these differences were largely mitigated after MRD-based treatment intensification. This finding suggests that MRD-guided therapy may help overcome the prognostic limitations of traditional early morphological response assessments. Patients initially classified as having SER, who received treatment intensification based on MRD, showed outcomes comparable to those of RER patients [2, 4, 14, 15].

The evolution of MRD monitoring approaches in our study reflects broader technological advances in the field. The transition from flow cytometry (sensitivity 10^-4^) to NGS (sensitivity 10^-6^) in August 2021 represents a considerable advancement in detection capabilities [7]. This improved sensitivity is important for identifying patients with low-level disease, which is potentially overlooked by less sensitive methods [16].

The impact of treatment intensification evolved during our study period, with implementation rates varying from 9.1% (up to 2015) to 28.6% (2016–2019) and 13.9% (2020–2023) (Supplementary Table 2). This trend may reflect growing confidence in MRD-based decision-making and the refinement of treatment protocols. Moreover, the reduced proportion in the most recent period may indicate more precise initial risk stratification and better upfront therapy selection. This finding was reflected in the progressive improvement of EFS over time (Supplementary Fig. 3b).

Different genetic subtypes showed distinct patterns of MRD clearance, providing insights into the molecular basis of treatment response. In contrast to the findings of Pui et al. [17], where *ETV6::RUNX1* and hyperdiploidy > 50 showed particularly favorable MRD clearance with a 5-year EFS of 91.4% and 89.6%, respectively, and Yu et al. [18], where favorable genotypes revealed substantially better outcomes with MRD-guided therapy, our cohort demonstrated similar survival benefits across molecular subtypes following MRD-guided intensification. Our analysis did not determine the underlying mechanism for these discrepancies, because our study protocol was not designed to prospectively evaluate MRD-based treatment intensification. These findings warrant further investigation in large-scale prospective trials using protocol-defined MRD-guided treatment strategies.

Our study had certain limitations. First, because of the retrospective nature of our study, the treatment intensification criteria were not prospectively standardized. Second, this study had a relatively small sample size. Third, the evolution of treatment protocols and MRD detection methods during the study period may have introduced variability in patient management. Fourth, decisions regarding intensification in patients with a high MRD were primarily influenced by the clinician’s judgment at the time of therapy. Clinicians tend to make individualized decisions based on each patient’s clinical circumstances. Therefore, our results should be interpreted with caution because of the potential variability introduced by the clinical decision-making process.

In conclusion, our findings strongly support the value of MRD-guided therapy intensification in the treatment of pediatric ALL. Dynamic assessment of MRD throughout treatment enables the development of more personalized therapeutic strategies, potentially reducing relapse rates and improving long-term survival. These findings support the continued integration of MRD monitoring into ALL treatment protocols, and highlight the need for further research to optimize MRD-based treatment decisions. Ongoing prospective multicenter trials with larger cohorts and standardized NGS-MRD assessment methods will further validate and refine these approaches for pediatric ALL treatment.

## Supplementary Information

Supplementary Material 1.

## Data Availability

No datasets were generated or analysed during the current study.
