# Supplementary material for: Improved survival in pediatric acute lymphoblastic leukemia through therapy intensification based on minimal residual disease and protocol-driven early response risk classification
Source: Blood Res. 2025 Jul 9;60(1):40. doi: 10.1007/s44313-025-00085-3 (PMC12240913; doi:10.1007/s44313-025-00085-3)

**Supplementary Information**

**Increased survival rate by the pediatric acute lymphoblastic leukemia therapy intensification based on minimal residual disease levels along with protocol-based risk classification by early response**

Hyery Kim^1^, Su Hyun Yoon^1^, Sunghan Kang^1^, Kyung-Nam Koh^1^, Ho Joon Im^1^, Chan-Jeoung Park^2^, Mi Young Kim^2^, Young-Uk Cho^2^, Sang-Hyun Hwang^2^, Seongsoo Jang^2^, Jong Jin Seo^1, 3^

^1^Department of Pediatrics, University of Ulsan College of Medicine, Asan Medical Center, Seoul, South Korea

^2^Department of Laboratory Medicine, University of Ulsan College of Medicine, Asan Medical Center, Seoul, South Korea

^3^Korean Association for Children with Leukemia and Cancer

Corresponding Author: Hyery Kim, M.D., Ph. D

Email: taban@hanmail.net

**Supplementary Table** **1**. **Treatment regimens included in this study**

| **Modified AALL0331** | | |
| --- | --- | --- |
| Phase | Treatment |  |
| Induction | Dexamethasone | 6 mg/㎡ #2 PO x 28 days, D1-28 |
|  | Vincristine | 1.5 mg/㎡ (Max. 2 mg) IV push, D1, 8, 15, 22 |
|  | L-asparaginase | 6,000 IU/㎡ IM x 9 doses beginning D4 or 5 or 6 |
|  |  | (PEG-asparaginase 2,500 IU/㎡ IM x 1 dose, D4 or 5 or 6) |
|  | IT Ara-C | 30 mg (1-1.99 yrs), 50 mg (2-2.99 yrs), 70 mg (≥ 3 yrs), D1 |
|  | IT MTX | 8 mg (1-1.99 yrs), 10 mg (2-2.99 yrs), 12 mg (3-8.99 yrs), 15 mg (≥ 9 yrs) |
|  |  | CNS 1 & 2 - D8, 29 / CNS 3 - D8, *15, *22, 29 |
| **Protocol based risk stratification** | **RER** | **M1 at day 8 or day 15** |
|  | **SER** | **㎡ or M3 at day 15** |
| Consolidation | 6-MP | 50 mg/㎡/day PO, D1-14 & D29-42 (OT/PT<5 x UNL) |
|  | Cyclophophmide | 1,000 mg/㎡ in D5W 100 ml/㎡ IV over 1 hr, D1 & 29 with mesna |
|  | Ara-C | 75 mg/㎡ IV push or SC, D1-4, 8-11, 29-32, 36-39 |
|  | Vincristine | 1.5 mg/㎡ (Max. 2 mg) IV push, D15, 22, 43, 50 |
|  | L-asparaginase | 6,000 IU/㎡ IM, total 6 doses beginning D15 + total 6 doses beginning D43 |
|  |  | (PEG-asparaginase 2,500 IU/㎡ IM x 1 dose, D4 or 5 or 6) |
|  | IT MTX | 8 mg (1-1.99 yrs), 10 mg (2-2.99 yrs), 12 mg (3-8.99 yrs), 15 mg (≥ 9 yrs) |
|  |  | CNS 1 & 2 - D1, 8, *15, *22 / CNS 3 - D1, 8 |
| Interim maintenance (Standard) | Dexamethasone | 6 mg/㎡ #2 PO, D1-5, 29-33 |
|  | Vincristine | 1.5 mg/㎡ (Max. 2 mg) IV push, D1 & 29 |
|  | MTX | 20 mg/㎡/dose PO, D1, 8, 15, 22, 29, 36, 43, 50 (OT/PT<5 x UNL) |
|  | 6-MP | 50 mg/㎡/day PO, D1-50 (OT/PT<5 x UNL) |
|  | IT MTX | 8 mg (1-1.99 yrs), 10 mg (2-2.99 yrs), 12 mg (3-8.99 yrs), 15 mg (≥ 9 yrs), D29 |
| Interim maintenance (Augmented)-1 | Vincristine | 1.5 mg/㎡ (Max. 2 mg) IV push, D1, 11, 21, 31, 41 |
|  | MTX | 100 mg/㎡/dose escalate by 50 mg/㎡/dose IV push, D1, 11, 21, 31, 41 |
|  |  | Discontinue excalation and resume at 80% of last dose if delay it necessary for myelosuppression or Gr3 mucositis |
|  | L-asparaginase | 6,000 IU/㎡ IM, 6 doses beginning D2 + 6 doses beginning D22 |
|  |  | (PEG-asparaginase 2,500 IU/㎡ IM D2 & 22) |
|  | IT MTX | 8 mg (1-1.99 yrs), 10 mg (2-2.99 yrs), 12 mg (3-8.99 yrs), 15 mg (≥ 9 yrs), D1, 31 |
| Delayed intensification (Standard) | Dexamethasone | 10 mg/㎡ #2 PO, D1-21 (Do not taper) |
|  | Vincristine | 1.5 mg/㎡ (Max. 2 mg) IV push, D1, 8, 15 |
|  | Doxorubicin | 25 mg/㎡ IV over 5 mins, D1, 8, 15 |
|  | L-asparaginase | 6,000 IU/㎡ IM x 6 doses, beginning D4 or 5 or 6 |
|  |  | (PEG-asparaginase 2,500 IU/㎡ IM D4 or 5 or 6) |
|  | Cyclophophmide | 1,000 mg/㎡ in D5W 100 ml/㎡ IV over 1 hr, D29 |
|  | Mesna | 200 mg/㎡ in D5W (≥ 10kg 50 ml, < 10kg 30 ml) IV over 15 mins, pre -15 mins & post HR3, 6, 9 |
|  | 6-MP | 50 mg/㎡/day PO, D29-42 (OT/PT<5 x UNL) |
|  | Ara-C | 75 mg/㎡ IV push or SC, D29-32 & 36-39 |
|  | IT MTX | 8 mg (1-1.99 yrs), 10 mg (2-2.99 yrs), 12 mg (3-8.99 yrs), 15 mg (≥ 9 yrs), D1, 29 |
| Delayed intensification (Augmented)-1 | Dexamethasone | 10 mg/㎡ #2 PO, D1-21 (Do not taper) |
|  | Vincristine | 1.5 mg/㎡ (Max. 2 mg) IV push, D1, 8, 15, 43, 50 |
|  | Doxorubicin | 25 mg/㎡ IV over 5 mins, D1, 8, 15 |
|  | L-asparaginase | 6,000 IU/㎡ IM, 6 doses beginning D4 or 5 or 6 + 6 doses beginning D43 |
|  |  | (PEG-asparaginase 2,500 IU/㎡ IM D4 or 5 or 6 and D43) |
|  | Cyclophophmide | 1,000 mg/㎡ in D5W 100 ml/㎡ IV over 1 hr, D29 |
|  | Mesna | 200 mg/㎡ in D5W (≥ 10kg 50 ml, < 10kg 30 ml) IV over 15 mins, pre -15 mins & post HR3, 6, 9 |
|  | 6-MP | 50 mg/㎡/day PO, D29-42 (OT/PT<5 x UNL) |
|  | Ara-C | 75 mg/㎡ IV push or SC, D29-32 & 36-39 |
|  | IT MTX | 8 mg (1-1.99 yrs), 10 mg (2-2.99 yrs), 12 mg (3-8.99 yrs), 15 mg (≥ 9 yrs), D1, 29, 36 |
| Interim maintenance (Augmented)-2 | Vincristine | 1.5 mg/㎡ (Max. 2 mg) IV push, D1, 11, 21, 31, 41 |
|  | MTX | 50 mg/㎡/dose less than Max. dose from previous escalated dose in IM-1. |
|  |  | escalate by 50 mg/㎡/dose IV push, D1, 11, 21, 31, 41 |
|  |  | Discontinue excalation and resume at 80% of last dose if delay it necessary for myelosuppression or Gr3 mucositis |
|  | L-asparaginase | 6,000 IU/㎡ IM, 6 doses beginning D2 + 6 doses beginning D22 |
|  |  | (PEG-asparaginase 2,500 IU/㎡ IM D2 & 22) |
|  | IT MTX | 8 mg (1-1.99 yrs), 10 mg (2-2.99 yrs), 12 mg (3-8.99 yrs), 15 mg (≥ 9 yrs), D1, 31 |
| Delayed intensification (Augmented)-2 | Dexamethasone | 10 mg/㎡ #2 PO, D1-21 (Do not taper) |
|  | Vincristine | 1.5 mg/㎡ (Max. 2 mg) IV push, D1, 8, 15, 43, 50 |
|  | Doxorubicin | 25 mg/㎡ IV over 5 mins, D1, 8, 15 |
|  | L-asparaginase | 6,000 IU/㎡ IM, 6 doses beginning D4 or 5 or 6 + 6 doses beginning D43 |
|  |  | (PEG-asparaginase 2,500 IU/㎡ IM D4 or 5 or 6 and D43) |
|  | Cyclophophmide | 1,000 mg/㎡ in D5W 100 ml/㎡ IV over 1 hr, D29 |
|  | Mesna | 200 mg/㎡ in D5W (≥ 10kg 50 ml, < 10kg 30 ml) IV over 15 mins, pre -15 mins & post HR3, 6, 9 |
|  | 6-MP | 50 mg/㎡/day PO, D29-42 (OT/PT<5 x UNL) (Do not give to pts receiving Cranial RT) |
|  | Ara-C | 75 mg/㎡ IV push or SC, D29-32 & 36-39 |
|  | IT MTX | 8 mg (1-1.99 yrs), 10 mg (2-2.99 yrs), 12 mg (3-8.99 yrs), 15 mg (≥ 9 yrs), D1, 29, *36 (Do not give to pts receiving Cranial RT) |
|  |  | * Omit D36 IT MTX and the 2 weeks of 6-MP (D29-42) for pts receiving cranial RT |
| Maintenance | Vincristine | 1.5 mg/㎡ (Max. 2 mg) IV push every 4 weeks, D1, 29, 57 |
|  | Dexamethasone | 6 mg/㎡ #2 PO 5 days every 4 weeks, D1-5, 29-33, 57-61 (Do not taper) |
|  | 6-MP | 50 mg/㎡/day PO, D1-84 (OT/PT<5 x UNL). Dose escalation during Maintenance. |
|  | MTX | 20 mg/㎡/dose PO weekly, D8, 15, 22, 29, 36, 43, 50, 57, 64, 71, 78 (OT/PT<5 x UNL). Dose escalation during Main. |
|  | IT MTX | 8 mg (1-1.99 yrs), 10 mg (2-2.99 yrs), 12 mg (3-8.99 yrs), 15 mg (≥ 9 yrs), D1 |
| **Korean multicenter study 0601 regimen** | | |
| Phase | Treatment |  |
| Induction | PD | 60 mg/㎡ #3 PO, D0-27, then taper over 2 wks |
|  | Vincristine | 1.5 mg/㎡ (Max. 2 mg) IV push, D0, 7, 14, 21 |
|  | Daunomycin | 25 mg/㎡ IV over 5 mins, D0, 7, *14, *21 |
|  |  | (*Day 14 & 21 ANC ≥500/μL, PLT ≥50K/μL / If Day 14 bone marrow shows M3, administer regardless of ANC) |
|  | L-asparaginase | 6,000 IU/㎡ IM x 3/wk, beginning D3, total 9 doses |
|  | IT Ara-C | 1-2 Yr 30 mg, 2-3 Yr 50 mg, ≥ 3 Yr 70 mg, D0 |
|  | IT MTX | 8 mg(1-1.99 yrs), 10 mg(2-2.99 yrs), 12 mg(3-8.99 yrs), 15 mg(≥ 9 yrs) |
| **Protocol based risk stratification** | **RER** | **M1 or ㎡ at day 8** |
|  | **SER** | **M3 at day 8, or extramedullary involvement, MLL rearrangement** |
| Consolidation | Cyclophosphamide | 1,000 mg/㎡ in D5W (≥ 10kg 100 ml, < 10kg 50 ml) IV over 30 mins, D0 & 28 with mesna |
|  | 6-MP | 50 mg/㎡/day PO, D0-13, D28-41 |
|  | Ara-C | 75 mg/㎡ IV or SC x 16 doses, D0-3, 7-10, 28-31, 35-38 |
|  | Vincristine | 1.5 mg/㎡ (Max. 2 mg) IV push, D14, 21, 42, 49 |
|  | L-asparaginase | 6,000 IU/㎡ IM, D14, 16, 18, 21, 23, 25, 42, 44, 46, 49, 51, 53 |
|  | IT MTX | 8 mg(1-1.99 yrs), 10 mg(2-2.99 yrs), 12 mg(3-8.99 yrs), 15 mg(≥ 9 yrs), D0, 7, 14, 21 |
| Interim maintenance (RER) | Vincristine | 1.5 mg/㎡ (Max. 2 mg) IV push, D0, 10, 20, 30, 40 |
|  | MTX | 100 mg/㎡(initial dose) IV push, D0, 10, 20, 30, 40 escalate by 50 mg/㎡/dose to toxicity |
|  | L-asparaginase | 15,000 IU/㎡ IM, D1, 11, 21, 31, 41 |
|  | IT MTX | 8 mg(1-1.99 yrs), 10 mg(2-2.99 yrs), 12 mg(3-8.99 yrs), 15 mg(≥ 9 yrs), D0 & 20 |
| Delayed intensification (RER) | Vincristine | 1.5 mg/㎡ (Max. 2 mg) IV push, D0, 7, 14, 42, 49 |
|  | Adriamycin | 25 mg/㎡ IV over 5 mins, D0, 7, 14 (ANC≥500/ul, PLT≥50K/ul) |
|  | L-asparaginase | 6000 IU/㎡ x 12 doses IM, (D3-14 ; 6 doses) & (D42-53 ; 6 doses) |
|  | Dexamethasone | 10 mg/㎡/day #3 PO, D0-6 & D14-20 (no taper) |
|  | Cyclophophamide | 1,000 mg/㎡ in D5W 100 ml/㎡ IV over 1 hr, D28 with mesna |
|  | 6-MP | 50 mg/㎡/day PO, D28-41 (T-bil>2 mg/dl hold) |
|  | Ara-C | 75 mg/㎡/day IV or SC, D28-31, 35-38 |
|  | IT MTX | 8 mg(1-1.99 yrs), 10 mg(2-2.99 yrs), 12 mg(3-8.99 yrs), 15 mg(≥ 9 yrs), D0, 28, 35 |
| Interim maintenance (SER) #1 | Vincristine | 1.5 mg/㎡ (Max. 2 mg) IV push, D0, 10, 20, 30, 40 |
|  | MTX | 100 mg/㎡(initial dose) IV push, D0, 10, 20, 30, 40 escalate by 50 mg/㎡/dose to toxicity |
|  | L-asparaginase | 15,000 IU/㎡ IM, D1, 11, 21, 31, 41 |
|  | IT MTX | 8 mg(1-1.99 yrs), 10 mg(2-2.99 yrs), 12 mg(3-8.99 yrs), 15 mg(≥ 9 yrs), D0 & 20 |
| Delayed intensification (SER) #1 | Vincristine | 1.5 mg/㎡ (Max. 2 mg) IV push, D0, 7, 14, 42, 49 |
|  | Adriamycin | 25 mg/㎡ IV over 5 mins, D0, 7, 14 (ANC≥500/ul, PLT≥50K/ul) |
|  | L-asparaginase | 6,000 IU/㎡ x 12 doses IM, (D3-14 ; 6 doses) & (D42-53 ; 6 doses) |
|  | Dexamethasone | 10 mg/㎡/day #3 PO, D0-6 & D14-20 (no taper) |
|  | Cyclophophamide | 1,000 mg/㎡ in D5W 100 ml/㎡ IV over 1 hr, D28 with mesna |
|  | 6-MP | 50 mg/㎡/day PO, D28-41 (T-bil>2 mg/dl hold) |
|  | Ara-C | 75 mg/㎡/day IV or SC, D28-31, 35-38 |
|  | IT MTX | 8 mg(1-1.99 yrs), 10 mg(2-2.99 yrs), 12 mg(3-8.99 yrs), 15 mg(≥ 9 yrs), D0, 28, 35 |
| Interim maintenance (SER) #2 | Same as IM #1 |  |
| Delayed intensification (SER) #2 | Vincristine | 1.5 mg/㎡ (Max. 2 mg) IV push, D0, 7, 14, 42, 49 |
|  | Daunorubicin | 25 mg/㎡ IV over 5 mins, D0, 7, 14 (ANC≥500/ul, PLT≥50K/ul) |
|  | L-asparaginase | 6,000 IU/㎡ x 12 doses IM, (D3-14 ; 6 doses) & (D42-53 ; 6 doses) |
|  | Dexamethasone | 10 mg/㎡/day #3 PO, D0-6 & D14-20 (no taper) |
|  | Cyclophophamide | 1,000 mg/㎡ in D5W 100 ml/㎡ IV over 1 hr, D28 with mesna |
|  | 6-MP | 50 mg/㎡/day PO, D28-41 (*Omit when Cranial RT) (T-bil>2 mg/dl hold) |
|  | Ara-C | 75 mg/㎡/day IV or SC, D28-31, 35-38 |
|  | IT MTX | 8 mg(1-1.99 yrs), 10 mg(2-2.99 yrs), 12 mg(3-8.99 yrs), 15 mg(≥ 9 yrs), D0, 28, **35 (Skip Day 35 if Cranial RT is given) |
|  | RT | Cranial or CSRT should begin by D29 of DI #2 therapy when ANC≥750/ul, PLT≥75K/ul |
|  |  | (all SER - Cranial RT(12Gy) / CNS3 - CSRT(cranial 18Gy, spinal 6Gy) /Exception : RER testicular ALL cases) |
| Maintenance | Vincristine | 1.5 mg/㎡ (Max. 2 mg) IV push every 4 weeks, D0, 28, 56 |
|  | PD | 40 mg/㎡ #3 PO 5 days every 4 weeks, D0-4, 28-32, 56-60 (Do not taper) |
|  | 6-MP | 50 mg/㎡/day PO, D0-83 (Dose adjust ANC 750-1500/ul, PLT>75K/ul) |
|  | MTX | 20 mg/㎡/dose PO weekly, D7, 14, 21, (**28), 35, 42, 49, 56, 63, 70, 77 (**Omit when IT MTX is given) |
|  | IT MTX | 8 mg(1-1.99 yrs), 10 mg(2-2.99 yrs), 12 mg(3-8.99 yrs), 15 mg(≥ 9 yrs), D0 & *28 (Day 28 is administered up to 4 cycles only) |
| **Korean multicenter study 1501 regimen** | | |
| Phase | Treatment |  |
| Induction | PD | 60 mg/㎡ #3 PO, D0-27, then taper over 2 wks |
|  | Vincristine | 1.5 mg/㎡ (Max. 2 mg) IV push, D0, 7, 14, 21 |
|  | Daunomycin | 25 mg/㎡ IV over 5 mins, D0, 7, 14, 21 |
|  |  | (*Day 14 & 21 ANC ≥500/μL, PLT ≥50K/μL / If Day 14 bone marrow shows M3, administer regardless of ANC) |
|  | L-asparaginase | 6,000 IU/㎡ IM x 3/wk, beginning D3, total 9 doses |
|  | IT Ara-C | 1-2 Yr 30 mg, 2-3 Yr 50 mg, ≥ 3 Yr 70 mg, D0 |
|  | IT MTX | 8 mg(1-1.99 yrs), 10 mg(2-2.99 yrs), 12 mg(3-8.99 yrs), 15 mg(≥ 9 yrs) |
| **Protocol based risk stratification** | **RER** | **M1 or ㎡ at day 8 & M1 at day 15** |
|  | **SER** | **other than RER, extramedullary involvement, WBC≥100,000/uL** |
| Consolidation | Cyclophosphamide | 1,000 mg/㎡ in D5W (≥ 10kg 100 ml, < 10kg 50 ml) IV over 30 mins, D0 & 28 with mesna |
|  | 6-MP | 50 mg/㎡/day PO, D0-13, D28-41 |
|  | Ara-C | 75 mg/㎡ IV or SC x 16 doses, D0-3, 7-10, 28-31, 35-38 |
|  | Vincristine | 1.5 mg/㎡ (Max. 2 mg) IV push, D14, 21, 42, 49 |
|  | L-asparaginase | 6,000 IU/㎡ IM, D14, 16, 18, 21, 23, 25, 42, 44, 46, 49, 51, 53 |
|  | IT-Triple | Age-adjusted, D0, 7, 14, 21 |
| Interim maintenance (RER) #1 | Vincristine | 1.5 mg/㎡ (Max. 2 mg) IV push, D0, 10, 20, 30, 40 |
|  | MTX | 100 mg/㎡(initial dose) IV push, D0, 10, 20, 30, 40 escalate by 50 mg/㎡/dose to toxicity |
|  | L-asparaginase | 15,000 IU/㎡ IM, D1, 11, 21, 31, 41 |
|  | IT-Triple | Age-adjusted, D0, 20 |
| Interim maintenance (SER) #1 | Vincristine | 1.5 mg/㎡ (Max. 2 mg) IV push, D0, 28 |
|  | MTX | 5,000 mg/㎡, D0, 7, 14, 28, 42, with leucovorin |
|  | 6-MP | 50 mg/㎡/day PO, D0-56, daily |
|  | IT-Triple | Age-adjusted, D0, 28 |
| Delayed intensification #1 | Vincristine | 1.5 mg/㎡ (Max. 2 mg) IV push, D0, 7, 14, 42, 49 |
|  | Adriamycin | 25 mg/㎡ IV over 5 mins, D0, 7, 14 (ANC≥500/ul, PLT≥50K/ul) |
|  | L-asparaginase | 6,000 IU/㎡ x 12 doses IM, (D3-14 ; 6 doses) & (D42-53 ; 6 doses) |
|  | Dexamethasone | 10 mg/㎡/day #3 PO, D0-6 & D14-20 (no taper) |
|  | Cyclophophamide | 1,000 mg/㎡ in D5W 100 ml/㎡ IV over 1 hr, D28 with mesna |
|  | 6-MP | 50 mg/㎡/day PO, D28-41 (T-bil>2 mg/dl hold) |
|  | Ara-C | 75 mg/㎡/day IV or SC, D28-31, 35-38 |
|  | IT-Triple | Age-adjusted, D0, 28, 35 |
| Interim maintenance (RER) #1 | Same as IM-RER #2 |  |
| Interim maintenance (SER) #1 | Same as IM-SER #2 |  |
| Delayed intensification #2 | Vincristine | 1.5 mg/㎡ (Max. 2 mg) IV push, D0, 7, 14, 42, 49 |
|  | Daunomycin | 25 mg/㎡IV over 5 mins, D0, 7, 14 (ANC ≥500/ul, PLT ≥50K/ul) |
|  | L-asparaginase | 6,000 IU/㎡ x 12 doses IM, (D3-14 ; 6 doses) & (D42-53 ; 6 doses) |
|  | Dexamethasone | 10 mg/㎡/day #3 PO, D0-6 & D14-20 (no taper) |
|  | Cyclophophamide | 1,000 mg/㎡ in D5W 100 ml/㎡ IV over 1 hr, D28 with mesna |
|  | 6-MP | 50 mg/㎡/day PO, D28-41 (T-bil>2 mg/dl hold) |
|  | Ara-C | 75 mg/㎡/day IV or SC, D28-31, 35-38 |
|  | IT-Triple | Age-adjusted, D0, 7, 14, 21 |
| Maintenance (RER) | Vincristine | 1.5 mg/㎡ (Max. 2 mg) IV push every 4 weeks, D0, 28, 56 |
|  | PD | 40 mg/㎡ #3 PO 5 days every 4 weeks, D0-4, 28-32, 56-60 (Do not taper) |
|  | 6-MP | 50 mg/㎡/day PO, D0-83 (Dose adjust ANC 750-1500/ul, PLT>75K/ul) |
|  | MTX | 20 mg/㎡/dose PO weekly, D7, 14, 21, (**28), 35, 42, 49, 56, 63, 70, 77 (**Omit when IT MTX is given) |
|  | IT-Triple | Age-adjusted, D0, 28 (up to cycle 4) |
| Maintenance (SER) | Vincristine | 1.5 mg/㎡ (Max. 2 mg) IV push every 4 weeks, D0, 28, 56 |
|  | PD | 40 mg/㎡ #3 PO 5 days every 4 weeks, D0-4, 28-32, 56-60 (Do not taper) |
|  | 6-MP | 50 mg/㎡/day PO, D0-83 (Dose adjust ANC 750-1500/ul, PLT>75K/ul) |
|  | MTX | 20 mg/㎡/dose PO weekly, D7, 14, 21, (**28), 35, 42, 49, 56, 63, 70, 77 (**Omit when IT MTX is given) |
|  | IT-Triple | Age-adjusted, D0 |


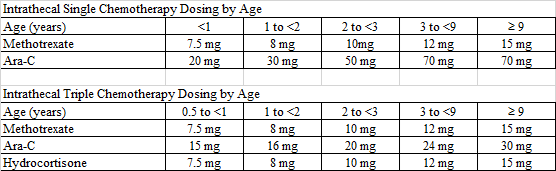


Abbreviations: ALL, acute lymphoblastic leukemia; PO, per os; IV, intravenous; IM, intramuscular; SC, subcutaneous; IT, intrathecal; ITT, intrathecal triple; MTX, methotrexate; Ara-C, cytarabine; 6-MP, 6-mercaptopurine; PD, prednisolone; CNS, central nervous system; RT, radiotherapy; CSRT, craniospinal radiotherapy; RER, rapid early responder; SER, slow early responder; ANC, absolute neutrophil count; PLT, platelets; WBC, white blood cell; PEG, polyethylene glycol; D5W, 5% dextrose in water; OT, SGOT (serum glutamic oxaloacetic transaminase); PT, SGPT (serum glutamic pyruvic transaminase); UNL, upper normal limit; HR, hour; T-bil, total bilirubin

**Supplementary Fig. 1** Flowchart summarizing patient classification, minimal residual disease Status, and treatment intensification in the analytic cohort


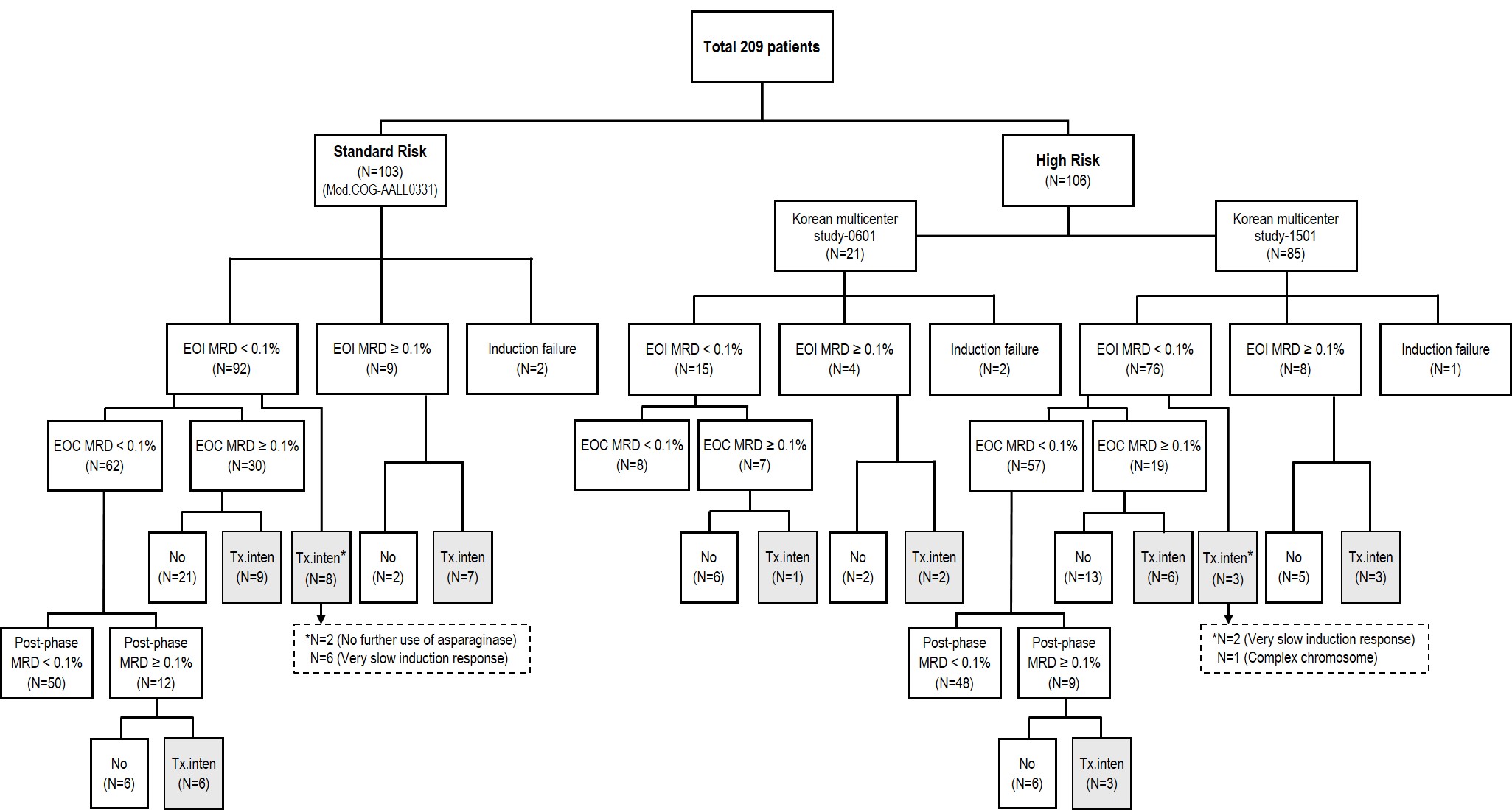


**Supplementary Table 2. Results of minimal residual disease assessment**

| **High MRD during the overall period** | | | | | | | | | | |
| --- | --- | --- | --- | --- | --- | --- | --- | --- | --- | --- |
|  | No. of Total | | ~2015 (N = 55) | | 2016–2019  (N = 77) | | 2020–2023  (N = 72) | | Subtotal | (%) |
| SR | 101 | | 15 | | 22 | | 15 | | 52 | 51.5 |
| HR | 103 | | 14 | | 21 | | 14 | | 49 | 47.6 |
| Total | 204 | | 29 (52.7%) | | 43 (55.8%) | | 29 (40.3%) | | **101** | **49.5** |
| **EOI High MRD** | | | | | | | | | | |
|  | No. of Total | | ~2015 (N = 55) | | 2016–2019  (N = 77) | | 2020–2023  (N = 72) | | Subtotal | (%) |
| SR | 101 | | 2 | | 2 | | 5 | | 9 | 8.9 |
| HR | 103 | | 5 | | 2 | | 5 | | 12 | 11.7 |
| Total | 204 | | 7 (12.7%) | | 4 (5.2%) | | 10 (13.9%) | | **21** | **10.3** |
| **EOC High MRD** | | | | | | | | | | |
|  | No. of Total | | ~2015 (N = 55) | | 2016–2019  (N = 77) | | 2020–2023  (N = 72) | | Subtotal | (%) |
| SR | 101 | | 15 | | 15 | | 5 | | 35 | 34.7 |
| HR | 103 | | 12 | | 14 | | 6 | | 32 | 31.1 |
| Total | 204 | | 27 (49.1%) | | 29 (37.7%) | | 11 (15.3%) | | **67** | **32.8** |
| **Post-phase (after consolidation blocks) High MRD** | | | | | | | | | | |
|  | No. of Total | ~2015 (N = 55) | | 2016–2019  (N = 77) | | 2020–2023  (N = 72) | | Subtotal | | (%) |
| SR | 101 | 14 | | 22 | | 10 | | 46 | | 45.5 |
| HR | 103 | 14 | | 19 | | 13 | | 46 | | 44.7 |
| Total | 204 | 28 (50.9%) | | 41 (53.2%) | | 23 (31.9%) | | **92** | | **45.1** |
| **Post-phase (after consolidation blocks) High MRD Only** | | | | | | | | | | |
|  | No. of Total | ~2015 (N = 55) | | 2016–2019  (N = 77) | | 2020–2023  (N = 72) | | Subtotal | | (%) |
| SR | 101 | 1 | | 0 | | 1 | | 2 | | 2.0 |
| HR | 103 | 0 | | 2 | | 0 | | 2 | | 1.9 |
| Total | 204 | 1 (1.8%) | | 2 (2.6%) | | 1 (1.4%) | | **4** | | **2.0** |
| **Treatment intensification d/t high MRD** | | | | | | | | | | |
|  | No. of Total | ~2015 (N = 55) | | 2016–2019  (N = 77) | | 2020–2023  (N = 72) | | Subtotal | | (%) |
| SR | 101 | 1 | | 14 | | 7 | | 22  (RER 19, SER 3) | | 21.8 |
| HR | 103 | 4 | | 8 | | 3 | | 15  (RER 15) | | 14.6 |
| Total | 204 | 5 (9.1%) | | 22 (28.6%) | | 10 (13.9%) | | **37** | | **18.3** |
| **Proportion of treatment intensified patients among patients with high MRD** | | | | | | | | | | |
|  | No. of Total | ~2015 (N = 29) | | 2016–2019  (N = 43) | | 2020–2023  (N = 29) | | Subtotal | | (%) |
| SR | 52 | 1 | | 14 | | 7 | | 22  (RER 19, SER 3) | | 42.3 |
| HR | 49 | 4 | | 8 | | 3 | | 15  (RER 15) | | 30.6 |
| Total | **101** | 5 (17.2%) | | 22 (51.2%) | | 10 (34.5%) | | **37** | | **36.6** |

Abbreviations: EOI, end of induction; EOC, end of consolidation; MRD, minimal residual disease; SR, standard risk; HR, high risk; RER, rapid early responder; SER, slow early responder

**Supplementary Fig. 2** Five-year event-free survival rates using flow cytometric MRD grades

1. Event-free survival by the end of induction (EOI) MRD


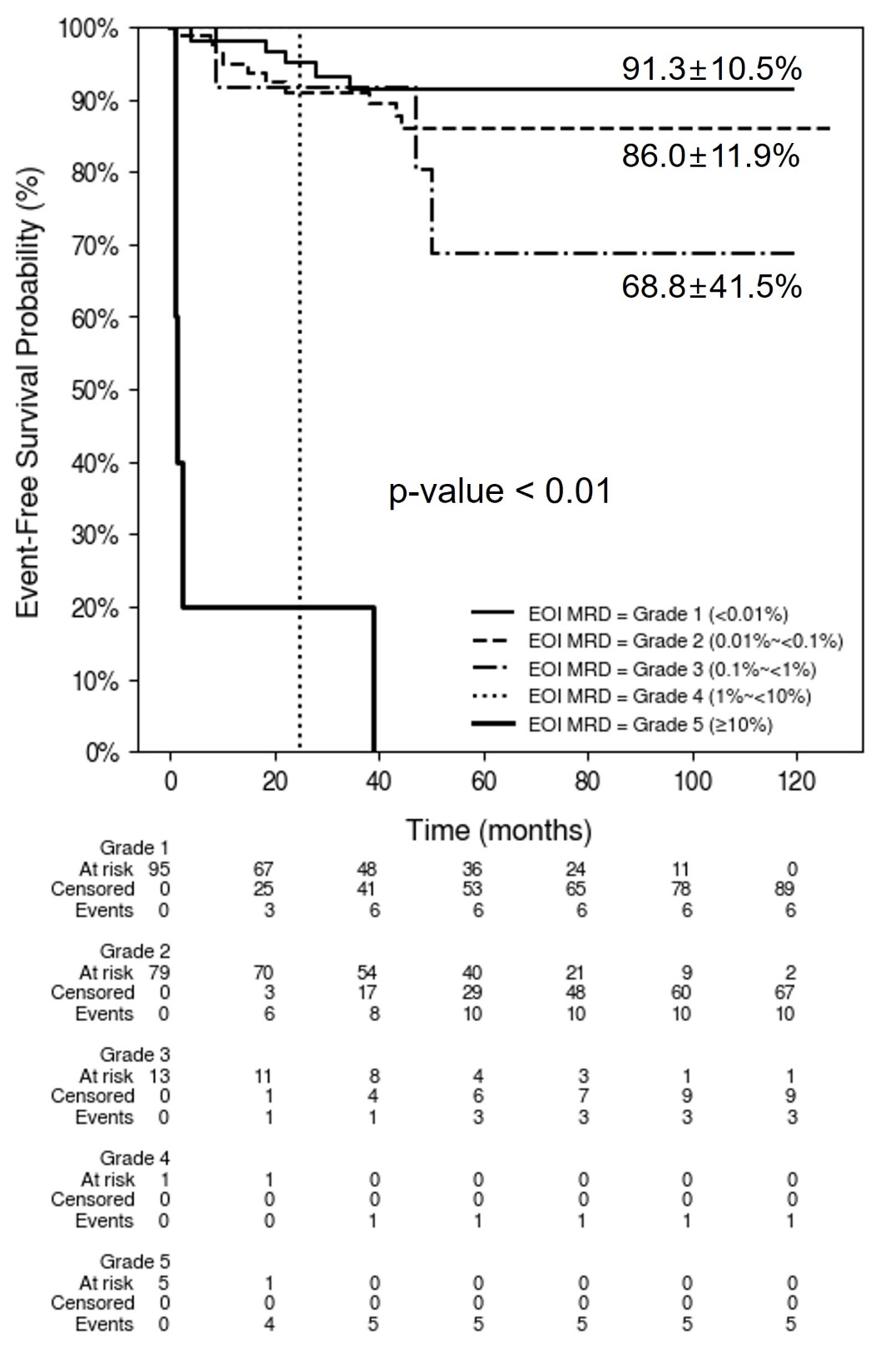


1. Event-free survival by end of consolidation (EOC) MRD


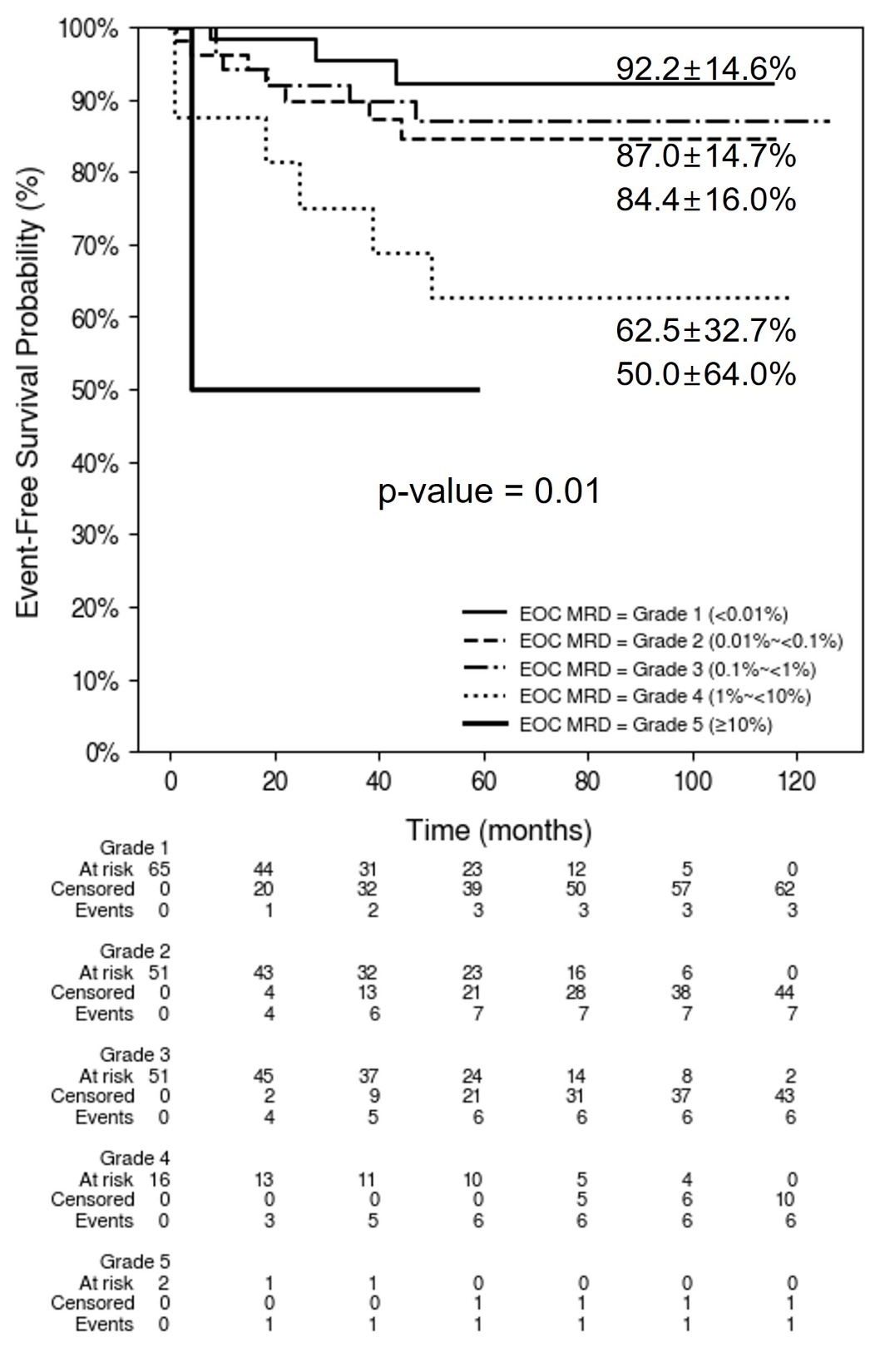


**Supplementary Table 3. Characteristics of the patients by treatment intensification**

|  | | No intensification | | Treatment intensification | | *P* |
| --- | --- | --- | --- | --- | --- | --- |
|  |  | N=63 | | N=38 | |  |
| Age | median (range, years) | 5.2 (1.3~17) | | 5.4 (1.3~14.6) | | 0.06 |
| WBC | median (range, /uL) | 22,600 (600~357,900) | | 9,900 (500~441,000) | | 0.16 |
| Sex | F | 28 | 44.4% | 23 | 60.5% | 0.12 |
|  | M | 35 | 55.6% | 15 | 39.5% |  |
| Immunophenotype | B-cell | 58 | 92.1% | 37 | 97.4% | 0.27 |
|  | T-cell | 5 | 7.9% | 1 | 2.6% |  |
| Moleculargenetics | B lymphoblastic leukemia/lymphoma, NOS | 20 | 31.7% | 16 | 42.1% | 0.21 |
|  | B lymphoblastic leukemia/lymphoma with t(1;19)(q23;p13.3); *E2A::PBX1* (*TCF3::PBX1*) | 8 | 12.7% | 1 | 2.6% |  |
|  | B lymphoblastic leukemia/lymphoma with t(12;21)(p13;q22); *TEL::AML1* (*ETV6::RUNX1*) | 10 | 15.9% | 6 | 15.8% |  |
|  | B lymphoblastic leukemia/lymphoma with FUS::ERG | 0 | 0.0% | 1 | 2.6% |  |
|  | B lymphoblastic leukemia/lymphoma with hyperdiploidy | 19 | 30.2% | 10 | 26.3% |  |
|  | B lymphoblastic leukemia/lymphoma with iAMP21 | 0 | 0.0% | 0 | 0.0% |  |
|  | B lymphoblastic leukemia/lymphoma with t(v;11q23); MLL rearranged | 1 | 1.6% | 3 | 7.9% |  |
|  | T lymphoblastic leukemia/lymphoma, *STIL::TAL1* | 4 | 6.3% | 0 | 0.0% |  |
|  | T lymphoblastic leukemia/lymphoma, NOS | 1 | 1.6% | 1 | 2.6% |  |
| Risk group | Standard risk | 30 | 47.6% | 22 | 57.9% | 0.32 |
|  | High risk | 33 | 52.4% | 16 | 42.1% |  |
| Chemotherapy regimen | modified COG-AALL0331 | 30 | 47.6% | 22 | 57.9% | 0.5 |
|  | Korean multicenter study-0601 | 9 | 14.3% | 3 | 7.9% |  |
|  | Korean multicenter study-1501 | 24 | 38.1% | 13 | 34.2% |  |
| CNS status at diagnosis | 1 | 54 | 85.7% | 37 | 97.4% | 0.33 |
|  | 2 | 3 | 4.8% | 1 | 2.6% |  |
|  | 3 | 6 | 9.5% | 0 | 0.0% |  |

Abbreviations: NOS, not otherwise specified; COG, children’s oncology group

**Supplementary Fig. 3** Five-year survival rates by the years of diagnosis in all patients

a. Overall survival


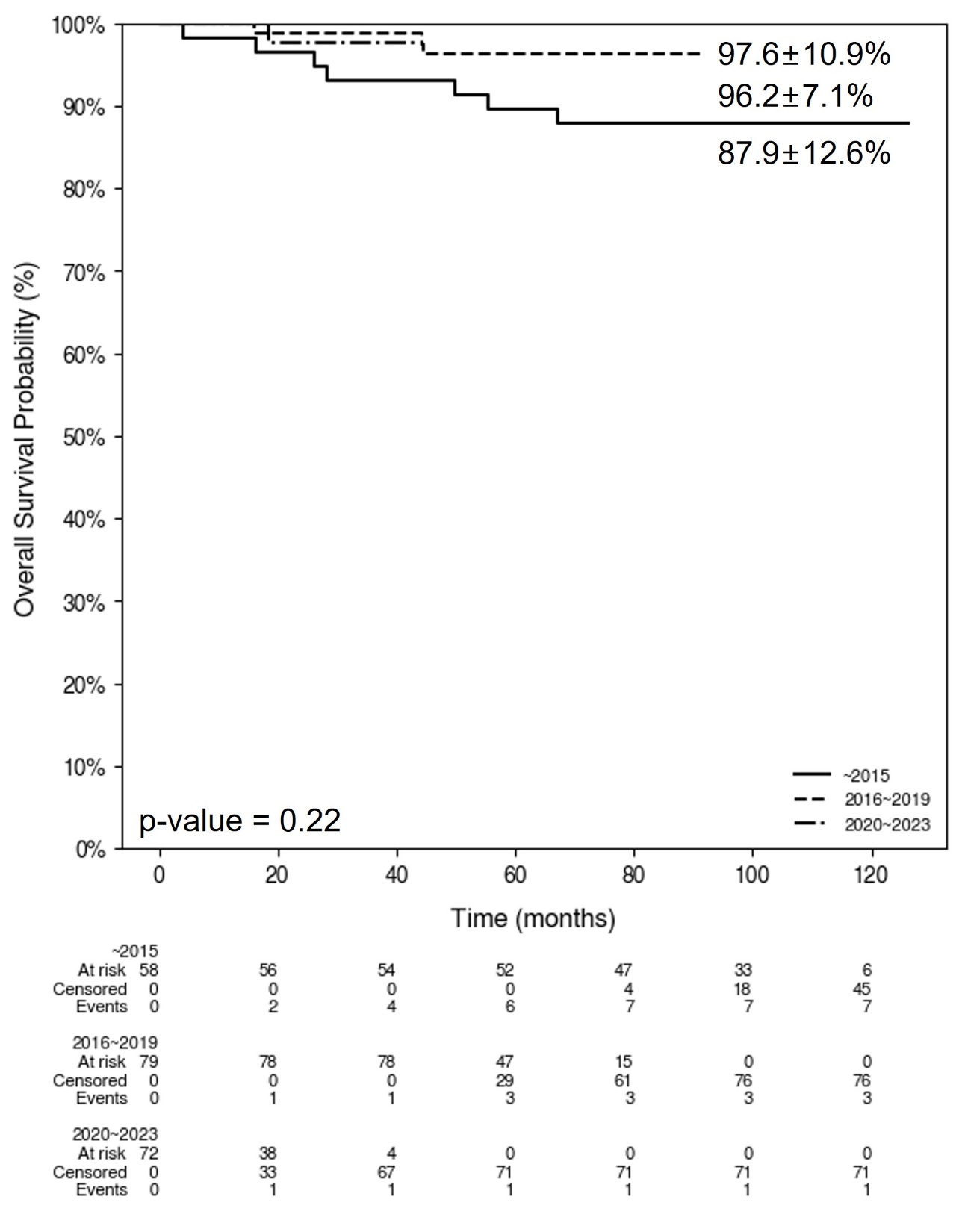


b. Event-free survival


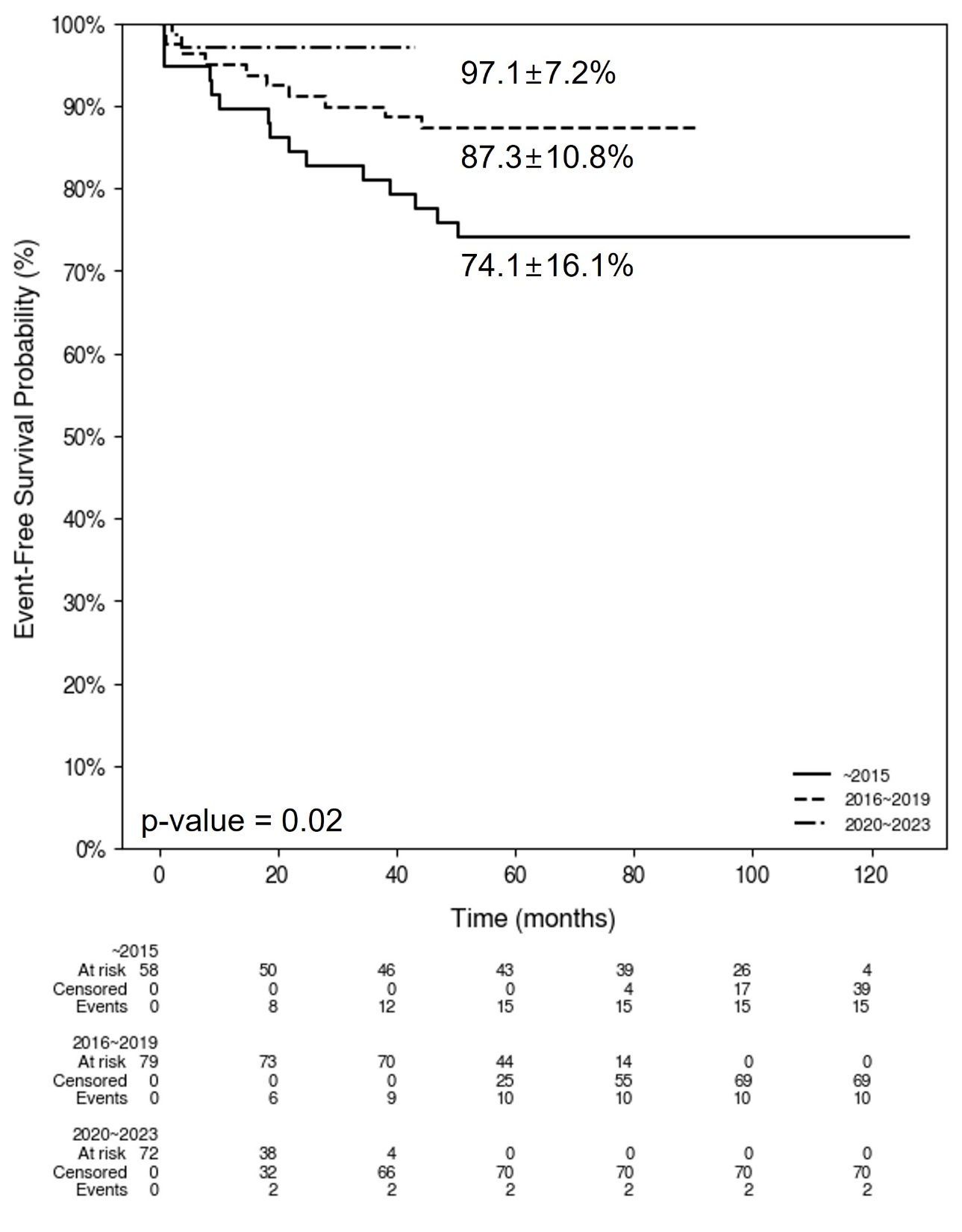

Supplement: Supplementary file 1 — Supplementary Material 1. [file 44313_2025_85_MOESM1_ESM.docx]
